# At-Home Stroke Neurorehabilitation: Early Findings with the NeuroExo BCI System

**DOI:** 10.3390/s25051322

**Published:** 2025-02-21

**Authors:** Juan José González-España, Lianne Sánchez-Rodríguez, Maxine Annel Pacheco-Ramírez, Jeff Feng, Kathryn Nedley, Shuo-Hsiu Chang, Gerard E. Francisco, Jose L. Contreras-Vidal

**Affiliations:** 1 Department of Electrical and Computer Engineering, University of Houston, Houston, TX 77004, USA; lsanch46@cougarnet.uh.edu (L.S.-R.); mpachec4@cougarnet.uh.edu (M.A.P.-R.); 2 Noninvasive Brain-Machine Interface Systems Laboratory, NSF Industry—University Cooperative Research Center for Building Reliable Advances and Innovations in Neurotechnology (IUCRC BRAIN) Center, University of Houston, Houston, TX 77004, USA; 3 College of Architecture and Design, University of Houston, Houston, TX 77030, USA; ffeng@central.uh.edu; 4 Department of Physical Medicine & Rehabilitation, University of Texas Health McGovern Medical School, Houston, TX 77030, USA; kathryn.nedley@memorialhermann.org (K.N.); shuo-hsiu.chang@uth.tmc.edu (S.-H.C.); gerard.e.francisco@uth.tmc.edu (G.E.F.); 5 Neurorecovery Research Center, TIRR Memorial Hermann, Houston, TX 77030, USA

**Keywords:** brain–computer interfaces, electroencephalography, stroke rehabilitation, movement intent detection, home therapy, wearables

## Abstract

Background: Democratized access to safe and effective robotic neurorehabilitation for stroke survivors requires innovative, affordable solutions that can be used not only in clinics but also at home. This requires the high usability of the devices involved to minimize costs associated with support from physical therapists or technicians. Methods: This paper describes the early findings of the NeuroExo brain–machine interface (BMI) with an upper-limb robotic exoskeleton for stroke neurorehabilitation. This early feasibility study consisted of a six-week protocol, with an initial training and BMI calibration phase at the clinic followed by 60 sessions of neuromotor therapy at the homes of the participants. Pre- and post-assessments were used to assess users’ compliance and system performance. Results: Participants achieved a compliance rate between 21% and 100%, with an average of 69%, while maintaining adequate signal quality and a positive perceived BMI performance during home usage with an average Likert scale score of four out of five. Moreover, adequate signal quality was maintained for four out of five participants throughout the protocol. These findings provide valuable insights into essential components for comprehensive rehabilitation therapy for stroke survivors. Furthermore, linear mixed-effects statistical models showed a significant reduction in trial duration (*p*-value < 0.02) and concomitant changes in brain patterns (*p*-value < 0.02). Conclusions: the analysis of these findings suggests that a low-cost, safe, simple-to-use BMI system for at-home stroke rehabilitation is feasible.

## 1. Introduction

Stroke-related costs in the United States reached almost USD 56.5 between 2018 and 2019. This figure includes healthcare services, stroke treatment medications, and the economic impact of missed workdays [1]. These costs are expected to grow largely due to the aging of the population [2]. This context highlights the need to develop affordable, easy-to-use, safe, and effective systems that promote at-home therapy solutions. In the case of upper-limb rehabilitation, some of the methods proposed in the literature include robotic rehabilitation [3], home-based telerehabilitation [4], video games [5], functional electrical stimulation [6], brain stimulation, and brain–computer/machine interfaces (BCI/BMI) [7], and virtual reality [8]. However, these approaches mostly focus on restoring arm strength. A holistic approach includes also the engagement, motivation, and reward of the participant, which are important factors for inducing cortical plasticity [9]. Building on previous approaches, combining robotic rehabilitation with a BCI/BMI system for closed-loop rehabilitation is anticipated to yield improved outcomes [9]. Furthermore, by incorporating home-based rehabilitation, the proposed system has the potential to democratize access to advanced rehabilitation technologies.

Bhagat et al. describes in [10] the development of an upper-limb stroke robotic rehabilitation BCI system using movement intent based on the Movement Related Cortical Potential (MRCP). In that study, the main finding was that MRCPs correlated with the efficacy of BCI-enabled robot-assisted rehabilitation in participants with chronic stroke, with 80% attaining minimal clinically important differences (MCID). Based on the results from that initial study, an affordable, easy-to-use, mobile, dry-electrode headset was proposed in [11,12] for deployment at home.

Here, we report the early findings of a clinical trial of the use of a robotic exoskeleton with the NeuroExo BMI system at home from a cohort of five stroke survivors. The focus was on usability, compliance, signal quality, perceived BCI performance, the evolution of MRCPs over time, and users’ feedback that led to device refinements. The rest of this paper is organized as follows: Section 2 describes the Materials and Methods, including a description of the study participants and metrics used to assess compliance and system performance. Section 3 presents the Results, featuring an analysis of user errors, device failures, and factors influencing user compliance. Section 4 provides recommendations for the design of EEG-based BMI systems for neurorehabilitation at home.

## 2. Materials and Methods

This section describes the study population, the design of the early-feasibility experimental study, the clinical assessments, and the metrics used to assess the users’ and system’s performance. This is based on the study proposed in [9,10].

### 2.1. Study Participants and Study Protocol

This study was conducted by the Texas Institute for Rehabilitation and Research (TIRR) Memorial Hermann and the University of Houston under a protocol approved by the Institutional Review Board of the University of Houston (STUDY00003430) and the Committee for the Protection of Human Participants of UT Health under the name “HSC-MS-20-1287 - PFI-RP: Smart co-robot system for cost-effective patient-centered robotic rehabilitation”.

The inclusion criteria were as follows: male or female participants aged 18 to 65 years old, with mild-to-moderate unilateral stroke confirmed by brain CT or an MRI scan and manifested by a Glasgow Coma scale (GCS) score between 15 and 9 documented within 6 months; ability to perform 20 deg of active wrist/elbow movement for upper-limb robotic movement on the affected side; no planned alteration in lower/upper-extremity therapy/medication for muscle tone during the course of this study; an anticipated length of needed acute interdisciplinary rehabilitation of 30 days or more; and a mini-mental state examination (MMSE) greater than or equal to 24 to rule out cognitive impairment and normal or near-normal strength in one upper/lower extremity and appreciable weakness in the other upper/lower extremity. The exclusion criteria included a history of traumatic brain injury prior to the current episode and a severe neurologic or psychiatric condition preventing participation in rehabilitation and physical therapy activities (e.g., participants were unable or unwilling to receive instruction and effectively complete a simple assigned task as determined by MMSE < 24). Women and minorities were recruited as long as they met the inclusion criteria.

Seven participants with chronic stroke were screened for this study, one participant (S1) withdrew after completing the first session of the study, and participant S4 did not meet the inclusion and exclusion criteria. The remaining five participants were enrolled, and their demographics are shown in Table 1. All participants had ischemic strokes. The table also provides information that can have an impact on usability, engagement, compliance, and customer/technical service. Two of the participants were from out of state (California and Maryland); three participants had a job and were actively working at the time of the testing. Three participants received assistance from a family member or friend during the protocol, e.g., for donning and doffing the device. One participant had an extensive travel work schedule during the course of this study. The range of time from the onset of the stroke to the date of trial enrollment varied from 11 months to 95 months. Only one participant (S6) was affected in his dominant hand. Most participants were right-handed and impaired on their left side.

Table 2 depicts the clinical assessments for participants S2, S3, and S7. Unfortunately, participants S5 and S6 did not return for the post-assessment, so their data only show the pre-assessments.

**Figure 1:**
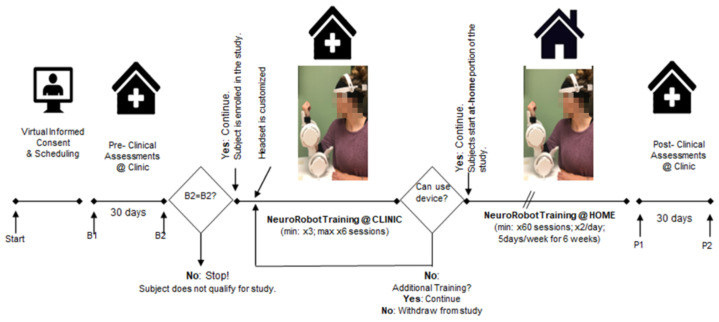
Timeline and phases of the early-feasibility testing for the NeuroExo BMI-exoskeleton system.

*Experimental Protocol:* Figure 1 depicts the timeline for the experimental protocol. After informed consent and a screening visit, two baseline clinical assessment visits were scheduled to confirm the absence of the spontaneous recovery of the participants. The assessments included the Fugl–Meyer Assessment for upper extremity (FMA-UE), the Manual Muscle Test (MMT), the grip strength test, the pinch strength test, the joint position sense test, the Jebsen–Taylor test, the usability scale, and the Action Research Arm Test (ARAT). If the participant did not show a change in any of the aforementioned metrics of more than 3 points, the participant was formally enrolled and proceeded to the next phase of this study, which involved up to 6 onsite training sessions at the clinic to receive instructions on donning/doffing the device and operating the device, including charging and setting up. During these sessions at the clinic, each participant performed up to 18 blocks, amounting to a total of 360 trials. The data from these trials were used for the calibration of the machine learning algorithm trained to detect movement intent from EEG signals, which is based on the results from Bhagat et al. [9,10]. The entire calibration session with the device involved setting up the headset, robotic arm, and tablet. Once the device was turned on, the participant’s arm was positioned on the robotic arm, and a block was started. This block included recording the electrode impedance and completing 20 trials. Trials comprised of looking at a target on the screen, holding the robotic arm in place, and then moving the arm at any point after the target changed its color to red. The end-effector position was indicated by a cursor on the screen, which could move up or down depending on a pseudorandom generator. Participants were instructed not to use the red color as a cue to move the arm. Instead, he/she was told that he/she could move the arm at any moment after that point. The session concluded after the completion of 3 blocks (60 trials).

**Figure 2:**
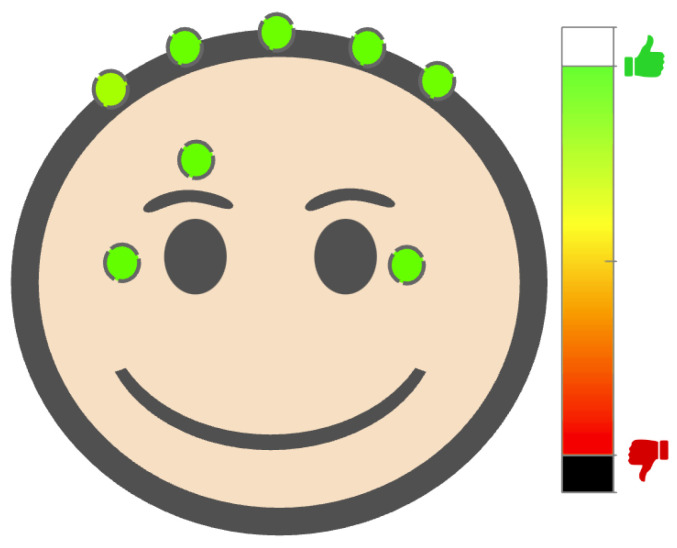
Graphics user interface (GUI) depicting the visual feedback provided to the user during the positioning of the NeuroExo device on the head. The impedance of EEG electrodes—scalp and EOG sensors—face are color-coded from low impedance (white) to high impedance (black) values. The correct positioning of the headset leads to lower impedance values.

**Figure A1:**
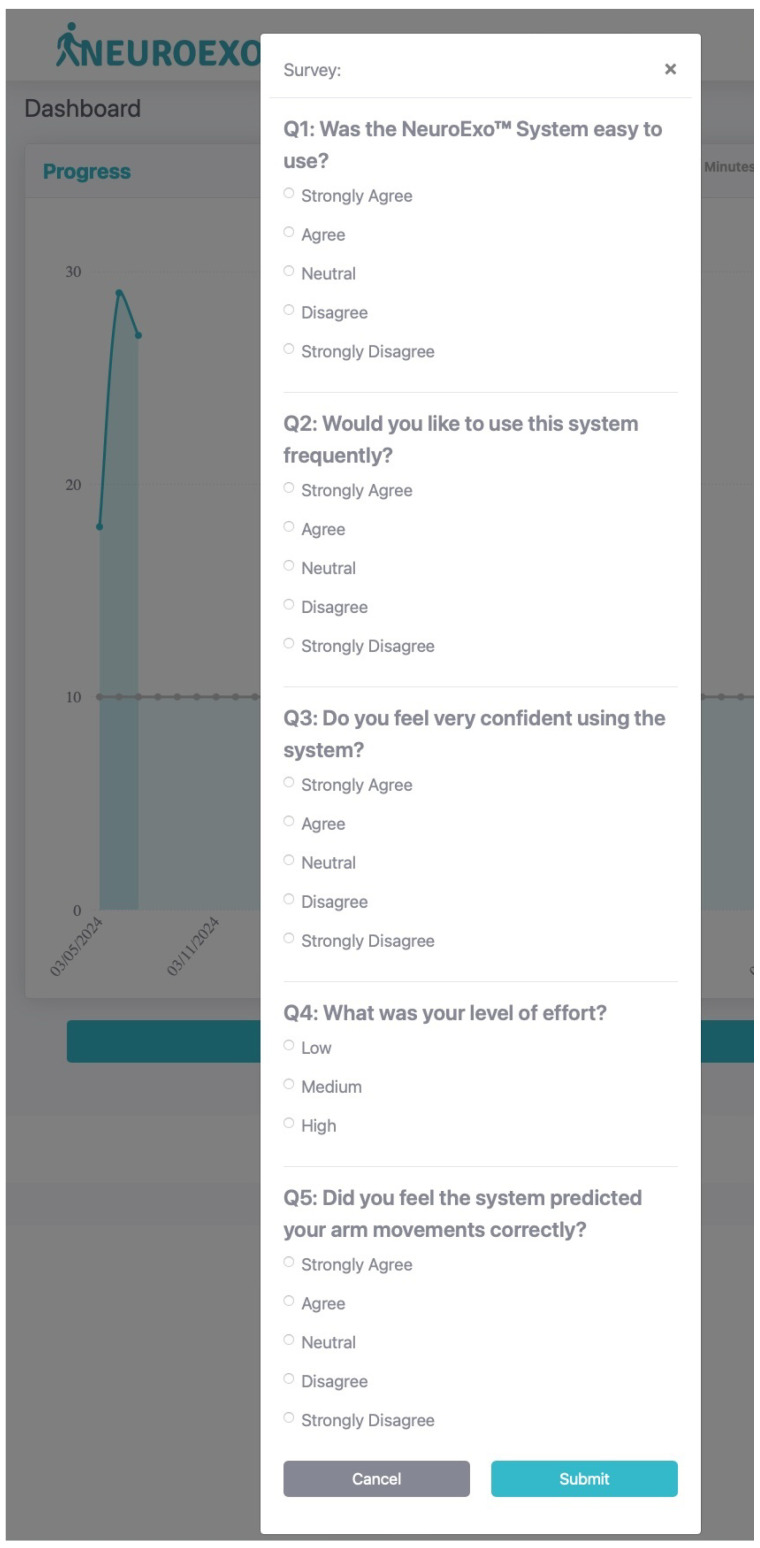
System assessment survey. Survey taken by participants after every session. It includes five prompts to assess usability, comfort, and perceived BCI performance of system.

After successfully completing the initial phase of this study at the clinic, the participant was provided with a NeuroExo system to take home. He/she was instructed to perform daily training with NeuroExo twice a day (morning and evening) for a total of sixty sessions of home neurotherapy. Sessions consisted of three blocks, with two sessions expected per day, resulting in a total of 10 sessions per week or 30 blocks per week. Each block comprised 20 trials, meaning that the weekly maximum was 10 sessions, 30 blocks, or 600 trials. During these sessions at home, the participants were required to follow the protocol without any assistance. To complete each experimental session participants, turned on the system, i.e., the robotic arm, headset, and tablet. Subsequently, they sat in a chair beside the robotic arm, secured themselves to it, and initiated the experimental session by interacting with the GUI. Once the block started, the system checked and recorded electrode–scalp contact impedance. The GUI displayed the quality of this contact, allowing the user to adjust the headset or individual electrodes to improve it (see Figure 2). Next, the system calibrated the built-in adaptive noise-canceling algorithm [13] to remove artifacts (eye movements, eye blinks, potential drifts, and biases) by asking the participant to blink three times. Then, the system allowed the participant to proceed with the first trial. The trials consisted of showing a target to the participant on the screen, and when the system detected the movement intent of the person three times in a row, based on the results of Bhagat et al. [9], the target turned “red” and the robotic arm assisted the participant to move the “neural” cursor up or down towards the target. Once all the session’s blocks were completed, each participant completed a survey that assessed usability and comfort (see Figure A1).

After the completion of the at-home phase of this study, each participant was invited back to the clinic for post-treatment (P1) and follow-up (P2) assessments to assess the level of motor recovery of the participants. These assessments included the FMA-UE, the MMT, and the grip, strength, and joint position sense tests, as in the pre-assessment phase.

The total duration of participation in the study was between 16 and 18 weeks.

### 2.2. NeuroEXO System

**Figure 3:**
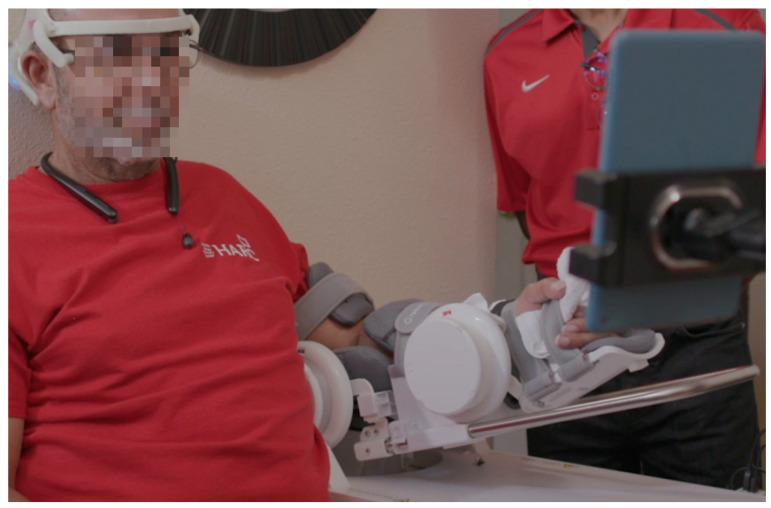
An example of a participant fitted with the NeuroExo device and upper-limb exoskeleton while performing a trial at home. The tablet allowed the participant to set up the system and receive visual feedback (reproduced with permission from [12]).

This study deployed the NeuroExo BMI headset previously described in [11,12] to control a robotic exoskeleton for the rehabilitation of the upper arm. Briefly, the NeuroExo headset consists of five dry-comb EEG electrodes, three eye sensors that measure electrooculography (EOG) activity, and an inertial measurement unit (IMU) to measure motion. All the BCI implementation is performed onboard using the Single Board Computer Beagle Bone Black-Wireless [14]. The BMI system includes adaptive noise-canceling (ANC) via the H-infinity denoising algorithm [13], a Support Vector Machine (SVM) trained on movement intent detection [9], the control of the robotic exoskeleton Rebless (H-robotics [15]), and the display of the graphical user interface (GUI) in the Amazon Fire Tablet [16]. Figure 3 shows a participant using the system.

### 2.3. Usability and Comfort

To measure the usability and comfort of the user with the system, a four-statement questionnaire was developed. The participants were asked to go to the survey section after each session, where they provided answers with the values “Strongly Agree”, “Agree”, “Neutral”, “Disagree”, and “Strongly Disagree”. The following questions were asked:

- **Q1:** Was the NeuroEXO^TM^ System easy to use?
- **Q2:** Would you like to use this system more frequently?
- **Q3:** Do you feel very confident using the system?
- **Q4:** What was your level of effort?

### 2.4. User and Performance Metrics

The following quantitative and qualitative metrics were used to characterize the Neuroexo system’s performance, compliance, and comfort:

#### 2.4.1. Electrode Impedance

The contact between the electrodes and the scalp was characterized by the electrical impedance measured in Ohms (Ω). A high impedance implied poor contact, while good contact was associated with low impedance. However, the acceptable impedance of the EEG electrodes depended on the input impedance of the amplifier. In the case of the NeuroExo system, the amplifier had an input impedance of 1 GΩ [12], allowing for higher electrode impedance values without significant signal quality loss. For this headset, the chosen acceptable impedance was associated with values below or equal to 100kΩ.

#### 2.4.2. Compliance Rate

For each week, the number of blocks was in the range [0,30] corresponding to the minimum and maximum usage of the system, respectively, and therefore an index of the users’ compliance. The compliance rate was determined by the participant completing 180 blocks required from this study.

#### 2.4.3. Block Rate

This metric is a numeric value that represents the approximate duration each participant spent per block.

#### 2.4.4. Perceived BCI Performance

This measures how participants perceived the BCI’s prediction of their intent to move their arm in each session. This was assessed by the question “Did you feel the system predicted your arm movements correctly?”, with five different options: “Strongly Agree”, “Agree”, “Neutral”, “Disagree”, and “Strongly Disagree”. It is important to note that this metric involved significant subjectivity, as it was based on the participants’ perception.

### 2.5. Movement-Related Cortical Potential (MRCP)

The MRCP is a cortical potential that precedes the voluntary movement of the upper limb. This has been used in [10] to predict the movement intent of a person and to calibrate the BCI. For each trial, the MRCP from electrode locations FC3, FC1, FCz, FC2, and FC4 were spatially averaged as defined by $X\left(t\right)=\frac{1}{5}\sum_{k=1}^{5}V_{k}\left(t\right)$, where $V_{k}\left(t\right)$ is defined as the EEG epochs of the trials [9], a method used for reducing noise in images. More details regarding MRCPs can be found in [17,18].

Every 50 ms, four features were calculated from the MRCP to estimate movement intent and were introduced into the SVM for prediction [12]. The features were the area, slope, negative peak amplitude, and Mahalanobis distance, defined as $d={\left[{\left(x-\mu \right)}^{T}\sum^{-1}\left(x-\mu \right)\right]}^{\frac{1}{2}}$. Three consecutive positive predictions of the SVM classifier were considered movement intent.

#### Analysis Software

The EEG data collected during the protocol were segmented into trials, filtered, and resampled by the NeuroEXO system. Before proceeding with analysis, any corrupt files were removed.

Offline data analysis was performed using custom code in Matlab 2023b, MATLAB’s Signal Processing and Statistics toolboxes [19], and the TDMS Reader library [20]. Trials were baseline corrected via the subtraction of the mean amplitude in the time window of interest with respect to movement-onset triggers as defined in [9]. Trials were spatially averaged, as defined in Section 2.5, across the channels of interest and plotted.

### 2.6. Device Troubleshooting and Users’ Errors

During the in-clinic training, the participants received a manual that included a troubleshooting section. It detailed expected system errors and their solutions. If a participant was not able to solve the problem based on the available steps, a technical call was initiated. In this call, an engineer guided the participant through additional steps that could resolve the problem. If the problem was solved during the technical support session, it was finished; otherwise, a home visit was arranged. Depending on the severity of the problem, it was either resolved in situ or taken to the technical lab for further troubleshooting. In the case of out-of-state participants, the system was shipped to the technical lab. After resolving the system error, either the same system or a new one was delivered to the participant’s home.

## 3. Results

In this section, the system performance, users’ feedback, users’ compliance, comfort, and the usability of the system are summarized based on the metrics described in Section 2.4. Additionally, reports detailing the users’ interactions with the system are presented, which guided the system’s refinement.

### 3.1. Signal Quality, Compliance, and Perceived BCI Performance

**Figure 4:**
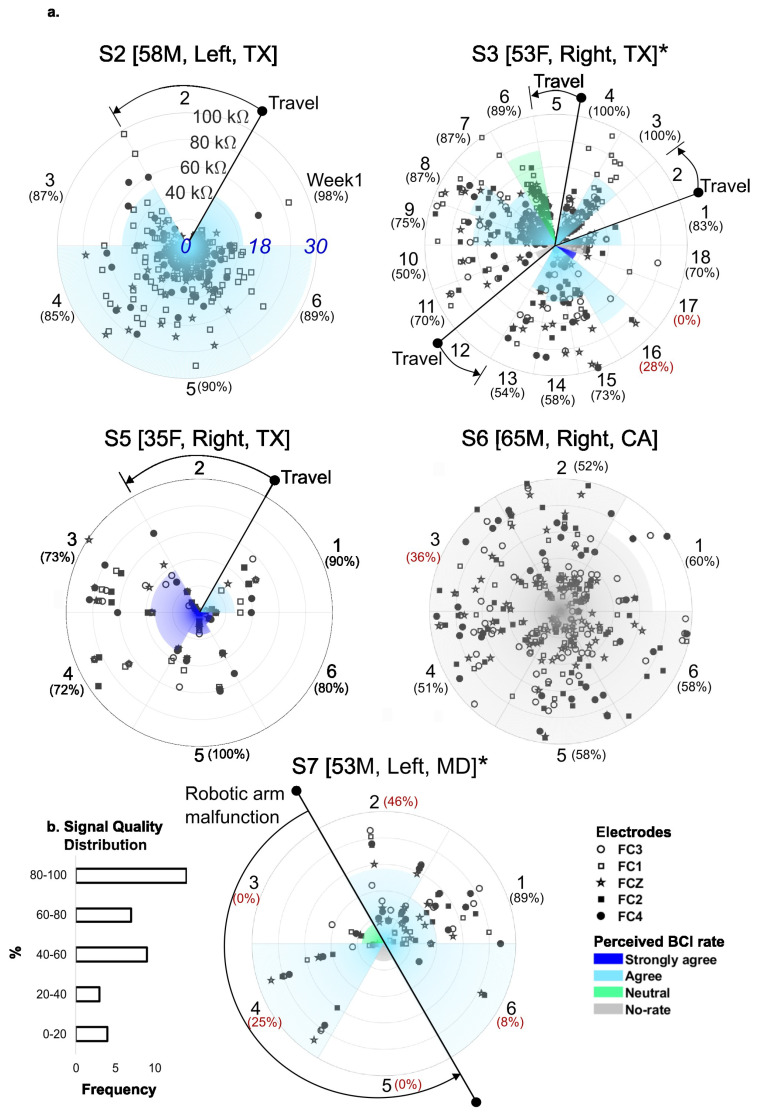
Characterizations of the performance of the NeuroExo system in terms of users’ compliance, perceived BCI performance, and electrode signal quality. (**a**). For each of the five participants with chronic stroke, the age, sex, impaired side, and home state are provided. Each graph depicts the level of electrode impedance [0, 100kΩ] (five symbols are used to code for electrode location along the frontocentral scalp in the 10–20 system). Users’ compliance is denoted as the number of blocks performed by the users per week in a counterclockwise direction (shading). The percentage of adequate impedance values (<=100kΩ) per week is shown in parenthesis. Note that participant S3 conducted NeuroExo therapy over 18 weeks due to therapy interruptions caused by extensive travel. Perceived BCI performance is color-coded by week on each graph. (**b**). The signal quality distribution is shown; the majority of the percentages for adequate impedances are located in upper buckets. Key: * indicates that these participants did not receive any assistance from family or friends during therapy.

Figure 4 depicts the users’ compliance, perceived BCI performance, and the signal quality of the recordings (i.e., electrode impedance) over the duration of this study, as defined in Section 2.4. In this figure, the green and red dots represent the average impedance for each week. Compliance is indicated by shading, with the color of the shading indicating the average perceived BCI performance. The participants with the highest compliance were S3 and S6, both at 100%, while the lowest was S5 at 21%. In terms of perceived BCI performance, all participants provided positive feedback, either agreeing or strongly agreeing that the BCI accurately predicted their movement intent. In general, all the participants, that is, S2, S3, S5, and S6, exhibited acceptable impedance performance, except for S7, with an impedance consistently above 100kΩ. Further details are discussed in the next section.

### 3.2. Device Usage

One of the main goals of this research was to identify and characterize any challenges encountered by the participants during their use of the NeuroExo system at their homes. These challenges could be characterized as participant-related (e.g., users’ errors) or system-related (e.g., device failure). Section 3.6 summarizes users’ challenges and device failures during the operation of the NeuroExo device.

#### 3.2.1. Participant S2

This participant was the first one enrolled in the protocol. His feedback allowed us to pinpoint areas for improvement in the firmware, GUI, and power system. For instance, during the second week, the participant encountered difficulties as the robotic arm did not move as expected due to robot malfunction, requiring technical support for a firmware upgrade. Because of this, he was not able to perform the therapy during that week (refer to Figure 3, showing a lack of data points during week 2). Two weeks later, the participant observed that the headset failed to hold a charge for more than two blocks, requiring a battery replacement. Overall, this participant showed high compliance to the therapy and found the NeuroExo to be of benefit and requested to continue using the device, which could not be honored due to the IRB protocol.

#### 3.2.2. Participant S3

Participant S3 took much longer to complete the protocol (18 weeks; see Figure 4). This was the result of multiple factors: First, the participant’s busy travel schedule disrupted the therapy timeline, so she could not perform the therapy in weeks 2, 5, and 12. Moreover, she also had difficulties using the micro-USB which led to redesigning the connector to USB-C to simplify use (she did not receive any assistance during the therapy). Additionally, in week 9, the robotic arm failed to recognize its paired device, resulting in a malfunction that prevented the trial from starting. This issue required a visit for re-pairing the devices and another visit to replace the robotic arm in week 11. Upon the analysis of this issue, the technical team considers it very likely that the nearby wireless devices affected the performance of the robotic arm.

#### 3.2.3. Participant S5

Participant S5 had the lowest compliance rate among all the participants with just 20.56%. This was the result of a busy work schedule during the study for this participant. No challenges with the system were reported.

#### 3.2.4. Participant S6

The out-of-state participant S6 did not report device malfunctions or other problems during this study. Unfortunately, the participant forgot to complete the surveys, a type of user error. This highlights the need to automatically prompt the survey after each session, rather than requiring the participant to search for it in the GUI.

#### 3.2.5. Participant 7

As described in Table 1, this participant lived in another state, and thus the technical team was limited to online or phone support while S7 experienced technical difficulties, such as the robotic arm not pairing. This participant presented a particularly challenging situation. Typically, to re-pair the robotic arm with the headset, an engineer would visit the participant’s home and use a specialized app provided by the manufacturer for troubleshooting. However, this app is not available on conventional platforms like the App Store or Google Play. Therefore, in this case, the manufacturer was required to provide special access for collaborators. In this case, this process necessitated coordination between the manufacturer, engineer, and participant. Due to the timing of this technical challenge, which occurred during the holidays, both the user and manufacturer had limited availability. As a result, resolving the issue took much longer than anticipated. Consequently, weeks 3, 4, and 5 of this study were significantly affected.

### 3.3. Trial Block Learning Rate

**Figure 5:**
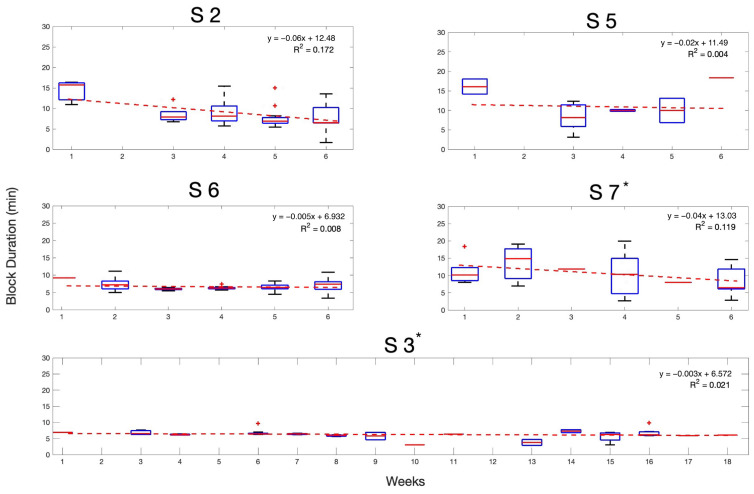
Trial block duration decreased with training. Boxplots display the distribution of impedance data across weeks and participants, with a linear fit overlaid to highlight trends in trial block duration (min) over time. Red + symbol indicates the outliers in the data. The fit was performed using MATLAB’s polynomial curve fitting function. Key: * indicates that these participants did not receive assistance from family members or friends during the trial.

Figure 5 shows the change in trial block duration as a function of week. It was expected that participants would become faster at performing the BCI task with training. Linear fitting on the data for each participant’s block duration indicated a learning process so that the block duration decreased by the end of this study (*p* < 0.05; one-tailed *t*-test), resulting in rejecting the null hypothesis (no downward change in trial block duration), thus indicating a decrease in the time that each participant spent in each trial block.

A linear mixed-effects (LME) model was employed for further statistical analysis, as LMEs accommodate participant-level variability effectively [21,22]. By using a linear mixed-effects model, it was possible to investigate the relationship between duration and weeks, while accounting for variability between the five participants.

The fixed effect of week, shown in Table 3, was found to be statistically significant (p=0.015), with an estimated decrease in duration of −0.182 units per additional week (95% confidence interval (CI): −0.329 to −0.036). The intercept, representing the initial average duration, was estimated at 9.426 units (95% CI: 7.791 to 11.061) and was also statistically significant (p<0.001).

The random effects analysis, shown in Table 4, also provided by the linear mixed-effects model showed that variability between participants had a standard deviation of 1.563 (95% CI: 0.765 to 3.193), while the residual standard deviation was 2.977 (95% CI: 2.687 to 3.297).

Overall, the model demonstrated a decrease in participants’ block durations across weeks, highlighting a significant relationship between weeks and block duration. While the model effectively captured the key trend of a decrease in block duration across weeks, the residual error suggests that incorporating additional data or variables could have further enhanced its ability to account for external conditions or individual differences among participants.

To further illustrate these trends, in addition to the linear trend representation, Figure 5 presents boxplots of trial durations across weeks for each participant. These visualizations show the block durations which overall show a decrease over time while accounting for variability across participants.

### 3.4. Blocks Analyzed

Specific blocks were chosen to evaluate early versus late MRCPs, AUC, and Mahalanobis distance changes. The features showcased in this paper were taken from manually chosen blocks that complied with specific criteria. The criteria that needed to be followed to be chosen for analysis were to have an impedance lower than 100 kΩ in the regions of interest, FC1, FCz, and FC2. Additionally, it was preferable that the two blocks used for analysis were taken on the same day and week; there were exceptions to the rule given the first criteria which will be discussed.

Table A1 demonstrates for each participant, in early versus late measurements, the week and date of the blocks chosen to analyze the results. For participant S2, early blocks were located in week 3, given that for week 1 and week 2, the robotic arm malfunctioned, causing data to be corrupted and unusable, as discussed in Section 3.2.1. Participant S3’s early blocks were taken from week 1, and late blocks were taken from week 13 to ensure good signal quality, given that the last weeks had a higher impedance. For participant S5, early and late blocks were taken from weeks 1 and 5, respectively; no data were analyzed from week 6 because FC1 showed a higher impedance. Participant S6’s data was extracted from weeks 1 and 6 accordingly. Finally, participant S7’s early measurements were taken on week 1; however, for late scenarios, one of the blocks was taken from week 3 and the second from week 6. This was due to the lack of good-quality signal regarding the impedance in the blocks during trials in weeks 3, 4, 5, and 6.

### 3.5. Changes in Brain Activity

**Figure 6:**
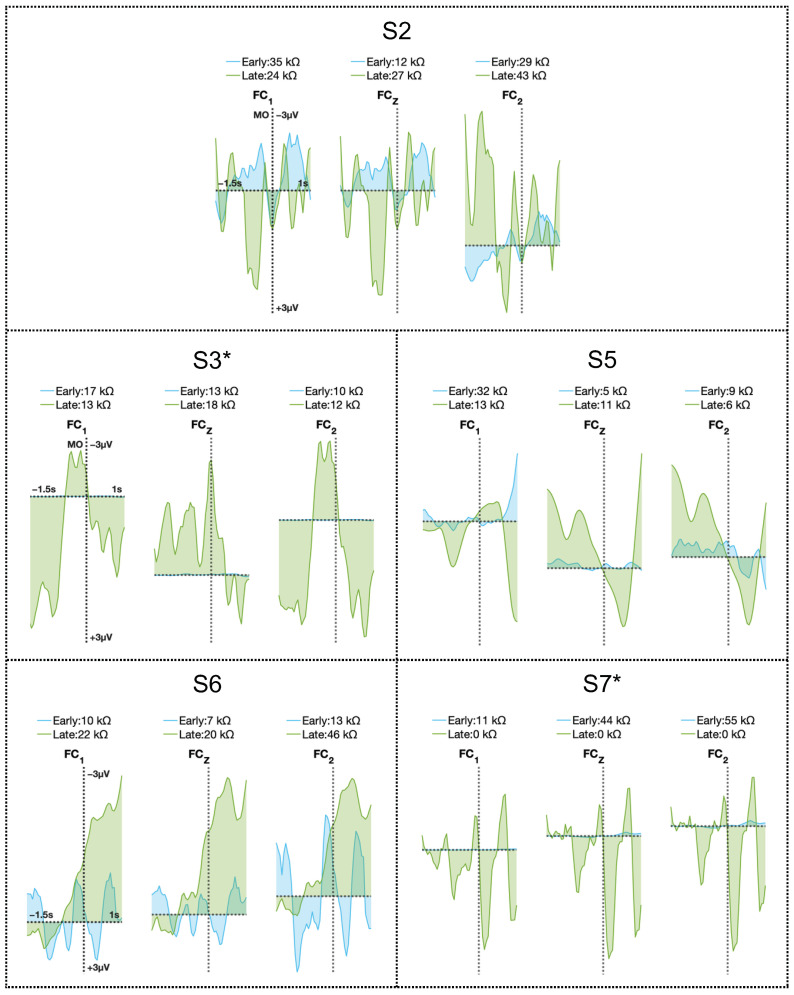
MRCP amplitude in early versus late sessions. Early MRCPs in blue represent the MRCP across a block of trials at the beginning of this longitudinal study and green MRCPs represent the last block of trials at the end of the longitudinal study. The annotation of the impedance values is provided to assess signal quality. Key: * indicates that these participants were not assisted by family members/friends.

**Figure 7:**
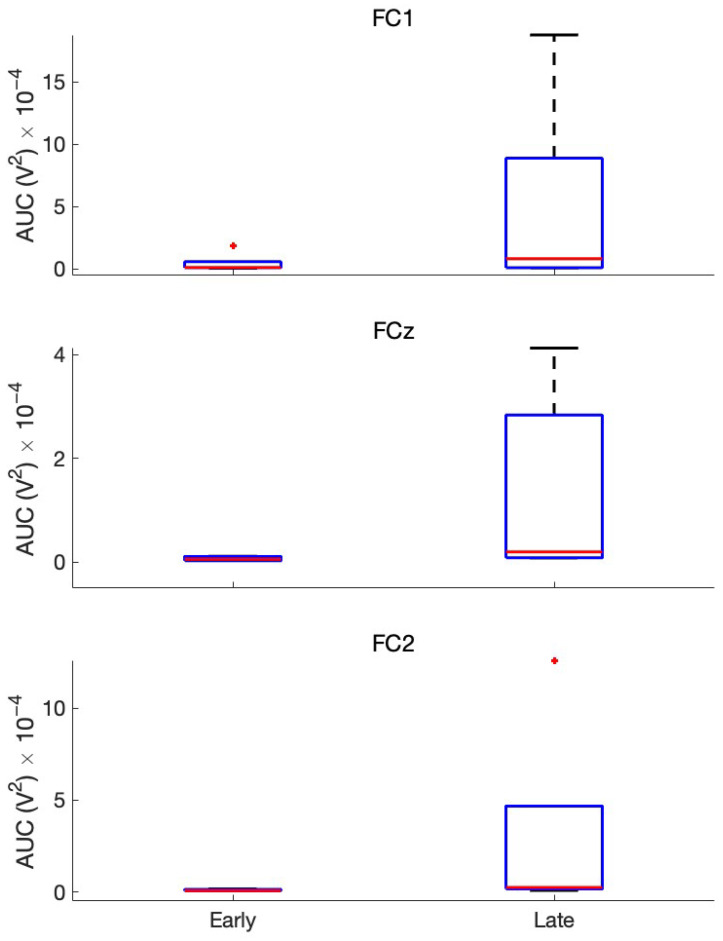
AUC amplitude in FC1, FCz, and FC2 in early versus late sessions. Each graph shows the early versus late Area Under the Curve (AUC) computed from the first and last two blocks in this longitudinal study for every participant by channel location. Red + symbol indicate the outliers in the data.

The NeuroExo headset [11,12] targeted the primary motor cortex as it records EEG signals from electrodes FC3, FC1, FCz, FC2, and FC4; however, the frontocentral electrodes (FC1, FCz, and FC2) had the most consistent impedance values across the participants, as shown in Section 3.1. The associated MRCPs are provided in Figure 6 (see Section 2.4). Figure 6 shows that the MRCPs generally had a higher AUC in the late-trial block compared to the first block (early training). Figure 7 shows changes in the AUC of the MRCPs for participants and electrodes of interest.

Participant S2, with a compliance rate of 68%, exhibited a slight decrease in the average MRCP AUC, accompanied by a four-point improvement in FMA-UE scores relative to baseline, as detailed in Table 2. This participant had the longest time since diagnosis (91 months) and showed a marginal increase in the AUC for FC2 between early and late stages, consistent with the impaired left side. However, changes in MRCP AUC were confined to a shorter time span due to a robotic arm system failure in week 2. This system failure likely influenced the results, as comparisons were limited to mid- and late-protocol stages.

Participant S3 showed a compliance rate of 100% and the highest increase in the mean AUC across the channels presented. The increase in AUC was higher for FC1, which was consistent with the impairment of the participant’s right side. Participant S3’s time since diagnosis was 45 months (refer to Table 1). Furthermore, S3 had a three-point improvement, a non-significant change, in the FMA-UE motor score relative to the baseline. Notably, participant S3, despite demonstrating high compliance, completed the sessions over a prolonged period. This deviation resulted in the sessions being conducted more sporadically and with reduced intensity compared to the protocol’s intended schedule, owing to travel constraints and system-related issues, as detailed in Section 3.2.2.

Participant S5 had, on average, a slight decrease in the early versus late AUC. However, the participant had low compliance with the protocol with a score of 21%, as detailed in Section 3.2.3, and it had been 11 months since their injury. For this participant, FCz and FC2 had an increase in the AUC, consistent with the fact that the participant was left-side impaired and thus had a right-hemisphere lesion. This suggests that even low compliance with the protocol resulted in a change in brain activity patterns from the impaired side. FMA-UE scores could not be assessed as the participant did not complete the post-intervention evaluations.

Participant S6 showed a slight increase in average AUC and had a compliance score of 100% and it was 14 months since their injury. This participant was impaired on the right-side, left-hemisphere lesion, where the highest increment in the AUC was for FC1 and FCz. Participant S7 has a compliance of 55% and showed an average increase in the AUC of the MRCPs. The participant had the highest increase in FCz and FC2, consistent with the impaired side (left side). FMA-UE scores could not be assessed as the participant did not complete the post-intervention evaluations. Participant S7’s time since diagnosis was 22 months, and they had no significant changes in the FMA-UE motor score, with a one-point improvement. Participant S7 had a compliance rate of 55%, due to system failures. Even though this participant’s results were positive, better results could have been achieved with higher compliance, given that issues with the system hindered the protocol’s progress, as discussed in Section 3.2.5.

A linear mixed-effects model was employed to analyze the relationship between the AUC and week (early and late), while accounting for variability between the five participants. The fixed effect of week was found to be statistically significant (p=0.011), with an estimated increase in the AUC of $5.9084\times 10^{-5}$ units in early versus late scenarios (95% confidence interval (CI): $1.442\times 10^{-5}$ to 0.00010375). Overall, the model demonstrated an increase in participants’ AUC across early versus late weeks, highlighting a significant relationship between the AUC and weeks. Please see Table 5.

### 3.6. Device Troubleshooting and Users’ Errors

One objective of this study was to assess the usability and reliability of the NeuroExo device for use at the homes of the participants, including potential failure modes and users’ errors. Table 6 depicts the failure modes and users’ errors and challenges experienced by the participants during this study. The analysis of these failure modes is important for design changes to mitigate or eliminate such failures or errors prior to clinical trial. Table 6 shows that the two most common failure modes were related to the robotic exoskeleton not pairing with the NeuroExo headset. This occurred intermittently for participants S2, S3, and S7. The analysis of this problem led to the conclusion that other devices in the vicinity of the robotic system interfered with its operation. The manufacturer’s decision is to migrate to a Bluetooth connection, which is currently the only option they are offering to the market. The second most prominent failure mode was structural (reported by participants S3, S6, and S7). Despite participants being trained on the correct method for donning and doffing the system, the technical team found evidence of mishandling. Specifically, areas of the device that were not reinforced and were not intended for donning/doffing were used inappropriately, contrary to the given instructions. These areas must be reinforced to prevent this failure mode in the future. One participant reported problems in connecting the charger’s connector to the device which led to the redesign of such connector to a USB-C type. Another participant reported issues with the battery’s range, which was solved by using a portable power bank which could also be used to extend the range of the system.

**Figure 8:**
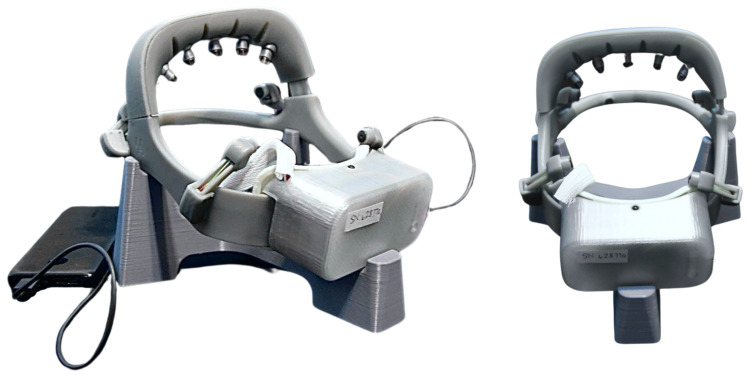
Newest NeuroExo headset version. Based on user feedback, some of the joints were reinforced, the micro-USB was replaced with USB-C, and the positioning of the EEG electrodes was more stable and easy to adjust.

The team noted that the travel and work schedules of two participants disrupted the usage of the NeuroExo system, which will need to be considered in a future clinical trial of the device. Figure 8 depicts the final version of the NeuroExo headset that was redesigned to address the device failure models reported above, including the reinforcement of parts that were affected by the handling of the device by the participants.

### 3.7. Participants’ Feedback

During this study, the authors received positive feedback from the users. In particular, two participants’ feedback stood out. Participant S2 mentioned that the system improved his arm strength and dexterity. He found the system easy to use. Furthermore, he expressed a desire to keep the system to continue using it at home, as he found the protocol beneficial for him.

Participant S7 commented that after the third week of the protocol, he regained mobility in his fingers. He also felt increased strength in his affected arm and experienced a significant overall improvement in arm movement.

No other feedback was provided by the other participants.

## 4. Discussion

From an electrical perspective, the Neuroexo headset generally delivered consistently adequate signal quality across four out of five participants, particularly for the frontocentral electrodes. However, several challenges became evident when implementing such systems within the population that were not initially taken into account as crucial factors. In terms of compliance, the outlier was one participant with just 21%, and the average was 69% among the five participants. Better results could have been achieved if the researchers were more proactive in confirming the progress of the participants and defining checkpoints that could have detected unperceived challenges encountered by the participants. Whilst this would have certainly improved compliance, it could have biased the participant to comply with the system because it could have been perceived as a responsibility rather than a rewarding experience. To identify the root cause of low compliance, one of the metrics that could be used is engagement. Whilst it is assumed that the low compliance of S5 was because of work schedule, it could also be that her being the youngest meant that the system was not engaging enough. Therefore, it is necessary to have engagement metrics that could factor out this variable or involve the participant in the GUI design process.

Furthermore, it is considered that technical challenges hampered usability and compliance results. Reported issues by the users included malfunction in the connection between the robotic arm and rest of the system, the complexity of plug in to the micro-USB connector, low battery endurance, server deadlock issues, and structural damage to the headset. Structural damage to the headset and connection malfunction interrupted protocol and data collection while the repair and/or replacement of them took place, thus affecting the compliance metric.

Regarding the BCI effectiveness, coming from feedback given by the participants, there was a positive response in which most of them agreed or strongly agreed that the headset accurately predicted their movement intent during the sessions. The BCI rate from the different participants suggests a learning effect, as demonstrated by the observed decrease in block duration over the weeks, notwithstanding having a small cohort of five participants. This trend indicates the participants’ increasing familiarity and proficiency with the experimental tasks, showing that as the weeks progressed, participants were likely to develop a better understanding of the protocol, leading to increased efficiency and faster completion of the block.

## 5. Conclusions

The focus of the current study was to assess the device usability and performance, users’ compliance, and users’ feedback during a longitudinal neurorehabilitation protocol using the NeuroExo system at the home of the participants. Using previously validated methods, the system was used by five stroke survivors for approximately 16–18 weeks. Information on usability, comfort, and impedance, was collected and analyzed. The BCI rate, compliance, perceived BCI performance, and the Fugl–Meyer score were also calculated to assess the system’s performance when used by the stroke survivor population. Participants achieved a compliance rate between 21% and 100%, with an average of 69%, while maintaining adequate signal quality in four out of five participants throughout the protocol. Moreover, perceived BMI performance during at-home sessions had an average Likert scale score of four out of five.

The results suggest that the type of connectors, location of reinforcement in the structure, and power system need special attention when the user is a stroke survivor or in other segments of the population with limited mobility. While the Fugl–Meyer scores (see Table 2) did not reach the threshold for statistically significant improvement, it is essential to develop metrics that offer a more comprehensive evaluation of participants. This is because, despite not meeting the threshold, the participants experienced improvements in their mobility. Furthermore, linear mixed-effects statistical models revealed a significant reduction in trial duration (*p*-value < 0.02), accompanied by concomitant changes in brain patterns (*p*-value < 0.02), further reinforcing the therapy’s impact.

Additionally, there is a need to conduct controlled trials for these systems, which is a challenge since participants will be aware if they are using the system, potentially influencing the results. While the NeuroExo headset was tested in a clinical application, the modularity of the system can be leveraged to extend its implementation to non-clinical BCI + IoT applications.

## Figures and Tables

**Table 1:** Participant demographics, clinical assessments, home state, work status (Y/N), helper (Y/N), travel status (Y/N), time since stroke (months), impaired side, and handedness.

ID (%)	Gender (M/F)	Age (Years)	Home State	Work (Y/N)	Help (Y/N)	Travel (Y/N)	Injury Dur (m.)	Impaired Side	Handedness (L/R)
S2	Male	58	TX	N	Y	N	91	L	R
S3	Female	53	TX	Y	N	Y	45	R	L
S5	Female	35	TX	Y	Y	N	11	L	R
S6	Male	65	CA	N	Y	N	14	R	R
S7	Male	53	MD	Y	N	N	22	L	R

**Table 2:** Clinical assessments for the experimental group. The symbols “✓” and “x” indicate whether the participant passed or did not pass the test, respectively.

Participant ID		S2				S3				S5		S6		S7		
Session		B1	B2	P1	P2	B1	B2	P1	P2	B1	B2	B1	B2	B1	B2	P1
NIH scale		5	4	2	4	2	0	2	2	7	7	7	6	5	4	6
FMA-UE	Motor	13	15	19	19	28	28	25	23	13	14	18	15	17	15	16
	Sensation	12	12	12	12	12	12	12	12	7	3	10	8	4	6	10
	Passive Joint Motion	21	24	22	21	21	21	23	22	24	22	22	24	20	19	18
	Joint Position	24	24	24	24	23	24	24	22	23	22	22	24	22	24	22
MMT	Elbow Flexor	4	4	4	4	5	5	5	NT	2	1	2	2	4	4	4
	Elbow Extensor	4	4	4	2	2	5	5	NT	0	0	0	1	1	2	1
	Wrist Flexor	0	1	2	3	5	5	2	NT	2	2	0	1	1	2	1
	Wrist Extensor	0	1	1	1	2	1	1	NT	0	0	0	1	0	1	0
	Finger Flexor	3	3	2	4	2	2	5	NT	1	3	2	0	2	3	1
	Thumb Flexor	4	4	4	2	4	4	4	NT	0	1	2	0	2	1	3
	Thumb Opponens	4	4	2	4	3	2	5	NT	0	0	2	0	1	2	3
Grip S.	lb	8.7	25.3	22.7	16.7	11.3	17	15	13	0	9.33	25.67	31	13	10.3	6.6
	% of Unimpaired	42	31.1	19.8	19.1	19.3	34	27.4	22	-	-	-	-	17	16.7	8.1
Pinch S.	lb	26.7	8.7	10	9.7	5.3	6	6	4	0	0.33	5	3.66	9.3	8.73	7
	% of the Unimpaired	24.5	40.1	40.6	40.4	32.5	40.8	36.8	24.5	-	-	-	-	38.8	38	25.6
Joint Position Sense	Metacarpohalangeal Joint	✓	✓	✓	✓	✓	✓	✓	✓	✓	x	✓	✓	x	x	✓
	Thumb Flex-Ext	✓	✓	✓	✓	✓	✓	✓	✓	x	x	i	✓	x	x	✓
	Wrist Flex-Ext	✓	✓	✓	✓	✓	✓	✓	✓	✓	i	✓	✓	x	✓	✓
	Elbow Flex-Ext	✓	✓	✓	✓	✓	✓	✓	✓	x	✓	✓	x	✓	✓	✓
Jebsen–Taylor	Writing	x	x	x	x	x	x	x	x	x	x	x	x	x	x	x
	Page Turing	x	x	x	x	60	53.98	53.71	60	x	x	x	x	x	x	x
	Lifting Small Objects	x	x	x	x	60	94	60	60	x	x	x	x	x	x	x
	Feeding	x	x	x	x	24.17	22.48	44.57	39.24	x	x	x	x	x	x	x
	Stacking	x	x	x	x	66.02	54.51	60	60	x	x	x	x	x	x	x
	Lifting Large Light Objects	x	x	x	x	60	47.19	60	41.62	x	x	x	x	x	x	x
	Lifting Large Heavy Objects	x	x	x	x	60	36.74	60	57.32	x	x	x	x	x	x	x
Usability Scale		-	-	62.5	-	-	-	55	-	-	-	-	-	-	-	50
ARAT		2	3	3	3	17	17	17	18	3	3	3	3	3	3	4

**Table 3:** Fixed-effects coefficients from the linear mixed-effects model.

Effect	Estimate	SE	t-Statistic	DF	*p*-Value	95% CI (Lower)	95% (Upper)
Intercept	9.426	0.829	11.371	187	<0.001	7.791	11.061
Week	−0.182	0.074	−2.450	187	0.015	−0.329	−0.036

**Table 4:** Random-effects covariance parameters from the linear mixed-effects model.

Group	Effect	Estimate	95% CI (Lower)	95% CI (Upper)
Participant	Intercept (std)	1.563	0.765	3.193
Residuals	Res Std	2.977	2.687	3.29

**Table 5:** Fixed-effects coefficients from the linear mixed-effects model for the early versus late AUC.

Effect	Estimate	SE	t-Statistic	DF	*p*-Value	95% CI (Lower)	95% CI (Upper)
Intercept	−0.0001	0.0001	−0.9435	28	0.3535	−0.0003	0.0001
Week	$5.908\times 10^{-5}$	$2.180\times 10^{-5}$	2.7097	28	0.0114	$1.44\times 10^{-5}$	0.0001

**Table 6:** A summary of system malfunctions encountered during the clinical trial. They are categorized into two types: system-related, corresponding to device failures, and participant-related, associated with the participant’s profile.

Issues	S2	S3	S5	S6	S7
Robotic arm not pairing	✓	✓			✓
Micro-USB charging port		✓			
Battery endurance	✓				
Server deadlock	✓				
Structural		✓		✓	✓
Heavy work schedule			✓		
No assistance		✓			✓

## Data Availability

Data are available upon reasonable request by contacting the corresponding authors.

## Abbreviations

The following abbreviations are used in this manuscript:

BCI	Brain–Computer Interface
IoT	Internet of Things
EEG	Electroencephalography
EOG	Electrooculography
MRCPs	Movement-Related Cortical Potentials
MoBI	Mobile Brain Interface
FM	Fugl–Meyer
SVM	Support Vector Machine
AUC	Area Under the curve
GUI	Graphical User Interface

## Appendix A

**Table A1:** Block information for analysis. Early and late blocks pairs are in same corresponding week except for late block in S7.

Participant	Protocol	Week	Date
S2	Early	3	1 July 2022
		3	1 July 2022
	Late	6	26 July 2022
		6	26 July 2022
S3	Early	1	22 August 2022
		1	22 August 2022
	Late	13	5 December 2022
		13	5 December 2022
S5	Early	1	20 September 2022
		1	20 September 2022
	Late	5	19 October 2022
		5	19 October 2022
S6	Early	1	10 November 2022
		1	11 November 2022
	Late	6	14 December 2022
		6	14 December 2022
S7	Early	1	7 October 2022
		1	7 October 2022
	Late	3	28 November 2022
		6	19 February 2023
